# The Effects of Parental Intervention on Sleep Patterns and Electronic Media Exposure in Young Adolescents

**DOI:** 10.3390/clockssleep4010013

**Published:** 2022-03-01

**Authors:** Ofra Flint Bretler, Orna Tzischinsky, Kfir Asraf, Tamar Shochat

**Affiliations:** 1 Department of Behavioral Sciences, Max Stern Academic College of Emek Yezreel, Jezreel Valley 1930600, Israel; ofraf@yvc.ac.il (O.F.B.); orna@yvc.ac.il (O.T.); 2 Faculty of Social Sciences, University of Haifa, Haifa 3498838, Israel; kfira@yvc.ac.il; 3 Faculty of Social Welfare and Health Sciences, University of Haifa, Haifa 3498838, Israel

**Keywords:** adolescence, electronic media, sleep patterns, parental intervention, parental training

## Abstract

Objective: This study evaluated the effectiveness of a parent-focused intervention aimed at the promotion of healthy sleep patterns and controlled exposure to electronic media (EM) in young adolescents. Participants: The sample included 70 dyads of parents (68 mothers and 2 fathers) and adolescents. Intervention and control groups each consisted of 35 young adolescents with a mean age of 10.7 (0.9) years old. Methods: Three waves of data collection included baseline, post-intervention, and 3 month follow-up. In each wave, adolescents reported habitual electronic media exposure and sleep patterns for a week and wore an actigraph for five nights. Parents in the intervention group participated in a six-session interactive workshop, while parents in the control group received equivalent written information by mail. Results: The intervention led to earlier bedtimes (*p* < 0.001), increased sleep efficiency (*p* < 0.01), increased sleep duration (*p* < 0.001) and reduced video games exposure (*p* < 0.01). Benefits were maintained at the follow-up. Conclusion: Interventions tailored for parents can create lasting positive changes in sleep patterns and EM exposure in young adolescents.

## 1. Statement of Significant

Exposure to electronic media has been associated with late bedtime and short sleep duration in adolescents. Interventions aimed to reduce screen time and promote better sleep habits have failed to establish long-term behavioral change. Evidence suggests that parental involvement contributes to improved sleep patterns. We assessed changes in sleep and electronic media use in adolescents following a six-session intervention for parents. Parents in the control group received written materials. Adolescents whose parents took part in the intervention had earlier bedtimes, longer sleep duration, and reduced time playing computer games at post-intervention and at a 3 month follow-up compared to baseline. These findings suggest that active parental involvement is a crucial ingredient for improving sleep and media habits in adolescents.

## 2. Introduction

In the past two decades, studies have demonstrated that increased screen time or use of electronic media (EM), including television, computer games, internet, and mobile phones, is a major contributor to poor sleep patterns [1,2]. In a systematic review dealing with the association between screen time and sleep outcomes among school-aged children and adolescents, screen time was found to be adversely associated with sleep outcomes, primarily shortened sleep duration and delayed bedtime [3,4]. Similar findings were reported in a meta-analysis that aimed to establish protective vs. risk factors for adolescent sleep disturbances [5]. Recent studies suggest that in addition to technology, higher soda intake and caffeine [6], tobacco, and alcohol, along with pre-sleep worry, are likely to have negative impacts on sleep [5]. On the other hand, parent-set bedtimes [5,7,8,9], internal bodily cues (using tiredness as a trigger for bedtime) [9], physical activity, and good sleep hygiene may be beneficial [5].

Several studies have examined “screen diet” interventions. An early study recruited participants in a substance abuse program who complained of sleep or daytime sleepiness [10]. Treatment included stimulus control therapy and the use of bright light to regularize sleep. Findings showed improved sleep and a reduction in substance abuse problems at the 12 month follow-up. The Food, Emotions, Routine, Restrict, Environment, and Timing (“FERRET”) intervention instructed adolescents to refrain from EM at least 30 min before bedtime, from exercise three hours before bedtime, and to perform no other activities in bed except for sleep [11]. Despite improved scores on sleep knowledge questions, there was no change in objective sleep duration. In another randomized trial of high school athletes, restricting EM after 10:00 p.m. did not improve sleep measurements [12]. In yet another study, restriction of mobile phone use one hour before bedtime for one school week increased total sleep time by 21 min; however, recruitment bias challenges the feasibility of such an intervention, as the adolescent population at large would likely not be motivated or committed to such a change in their mobile phone use [13].

Some sleep promotion programs have also been developed. For example, a one-week program that consisted of sleep education led to more regular bedtimes and shorter sleep-onset latency, with no improvements in sleep quality or daytime sleepiness [14]. Moseley and Gradisar [15] (2009) developed an intervention based on a cognitive behavior therapy framework. Despite a significant increase in sleep knowledge, no improvements were found in sleep measures. A subsequent intervention added elements of motivational interviewing, but again no behavioral changes were found [16]. The school-based “Sleep Smart Program” [17] was found to be effective in improving sleep health efficacy, sleep hygiene, time in bed, and bedtimes and in reducing internalizing behavior problems; however, changes were not sustained at follow-up [17]. In a recent study, adolescents with delayed sleep-phase disorder underwent bright light therapy sessions and advanced wake-up times, leading to advanced sleep onset, decreased sleep latency, and increased total sleep time [18].

Parental involvement in such interventions has scarcely been reported. In a recent review of recommendations to reduce the potentially problematic influence of screen time on pediatric sleep, the authors strongly recommended that clinicians work with families to reduce evening and nighttime EM exposure [4]. In a survey of Australian youth, only 17.5% of adolescents reported a parent-set bedtime as the main factor determining their bedtime on school nights [19]. Compared to adolescents without parent-set bedtimes, those with parent-set bedtimes had earlier bedtimes, achieved more sleep, and experienced improved daytime wakefulness and less fatigue. They were also significantly younger, as the percentage of adolescents whose parents set their bedtime steadily declined between the ages of 13 and 18. The authors concluded that parent-set bedtimes may offer a simple and easily translatable means for parents to improve the sleep and daytime functioning of their teens [19].

Despite evidence that parents can improve their adolescents’ sleep patterns, only one trial to date has included an educational program, parental involvement together with bright light therapy as part of an intervention for adolescents with habitual sleep problems [20]. The findings of this trial indicated an improvement in sleep knowledge and mood ratings and a decrease in sleep latency as compared to the control group that continued their regular classes as usual (and did not receive educational enrichment regarding sleep) [20]. Given the potential benefits of parental involvement, we developed a parental intervention targeting sleep and EM use, derived from an intervention model for children and adolescents aged 6 to 11 with eating disorders [21]. The model views parents as the sole agents of change in the lives of their children, and focuses on the influence of personal example, environmental changes, and the authoritative parental style [21]. The present feasibility pilot study aims to test the working hypothesis that parental involvement is a crucial ingredient towards the promotion of sleep health behaviors in young adolescents.

**Figure 1:**
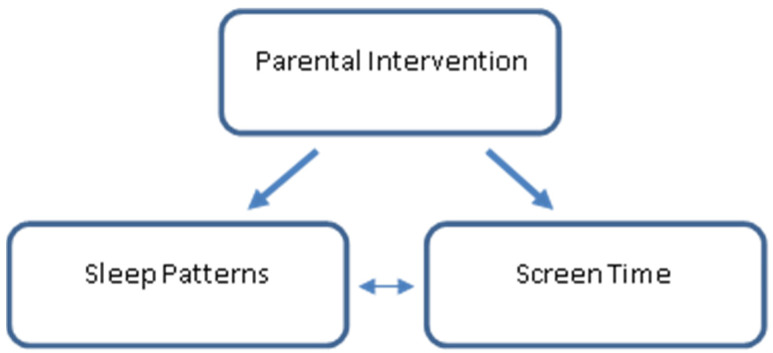
Theoretical model of this study.

According to the World Health Organization [9] (WHO) (Adolescents: Health Risks and Solutions, n.d.), adolescence begins at age 10. The literature divides adolescence and young adulthood as follows: “adolescence is divided into three developmental periods: early adolescence (10–14 years of age), late adolescence (15–19 years of age), and young adulthood (20–24 years of age)” [22]. Drawing conclusions from a parental intervention conducted on children and young adolescents with eating disorders [19] and from our previous results, adolescents between the ages 13 and 14 show inadequate sleep [2]. Alongside a decline in parental control over bedtime setting in later adolescence [19], we chose to focus on parents of young adolescents (ages 10–12). The main aim of this study was to evaluate the effectiveness of an interactive intervention program that focused on increasing parental knowledge of sleep, EM, health, and behavioral changes in adolescents. The intervention also gave parents tools to create environmental changes at home, provided solutions for difficult situations such as adolescent resistance, and discussed ways to raise the adolescents’ motivation to change. We hypothesized, that providing this information in the form of an interactive workshop, in comparison with providing the same information by written materials, would lead to improved sleep patterns (earlier bedtime, longer sleep duration, shorter sleep latency, and better sleep efficiency) and reduced EM exposure (television and computer use) in young adolescents (See Figure 1).

## 3. Results

### 3.1. Participants

Seventy dyads of parents (68 mothers and 2 fathers) and adolescents took part in the baseline assessment, 69 in the post-intervention assessment, and 68 in the 3 month assessment. Baseline assessments included 35 dyads in the intervention group and 35 dyads in the control group. The mean age of the adolescents was 10.69 ± 0.94, and the group included 35 boys (21 in the intervention group) and 35 girls (21 in the control group) [χ^2^(1) = 2.80, *p* = 0.094]. Parents’ mean age was 41.35 ± 3.92 years, their mean education level was 15.5 ± 2.36 years, and number of rooms in the house was 5.07 ± 1.02. Two mothers from the intervention group dropped out of this study, one at post-intervention and another during the follow-up (see flowchart).

### 3.2. Baseline Differences between Groups and by Sex

No differences found between the intervention and control groups in demographic characteristics (number of children, level of income, number of room in the house and year of education) or in sleep measures. Significant differences were found between the intervention and control groups on TV time on weekdays [t(62) = 2.04, *p* < 0.05], and on weekends [t(60) = 2.09, *p* < 0.05], so that the intervention group spent more time watching TV during the weekdays and the weekends. Sex differences were found only for computer games [t(35.68) = 3.43, *p* = 0.001], with boys (M = 2.46, SD = 2.26) spending more time on the computer than girls (M = 0.87, SD = 0.99).

### 3.3. Intervention

Within-between repeated-measures ANOVA analyses was performed to assess the difference in sleep patterns (SHSS and Actigraphy) and EM by time (baseline, post-intervention, and at the 3 month follow-up) and group (intervention/control).

### 3.4. Actigraphy

**Figure 2:**
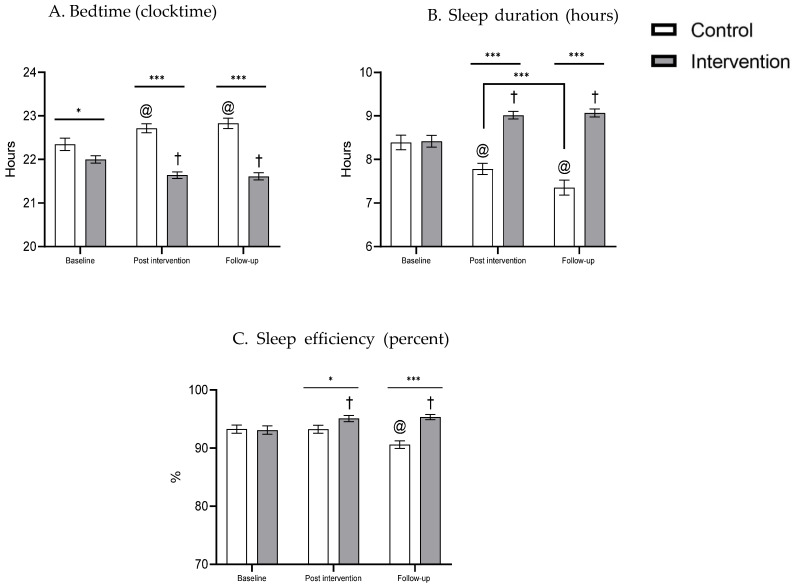
Actigraphy bedtime (**A**), sleep duration (**B**), and sleep efficiency (**C**) at baseline, post-intervention and follow-up, intervention and control groups. * *p* < 0.05, *** *p* < 0.001, † different from intervention baseline, @ different from control baseline.

The following effects were found for the actigraphy sleep measures (Table 1 and Figure 2):

Bedtime: A significant main effect was found for group [F(1, 66) = 54.53, *p* < 0.001], so that the intervention group went to bed earlier than the control group. While there was no main effect for time, a significant time X group interaction was found [F(2, 132) = 18.61, *p* < 0.001]. Post hoc analysis showed that the intervention group went to bed earlier than the control group at post-intervention [t(66) = −8.5, *p* < 0.001], and follow-up [t(60) = −8.32, *p* < 0.001]. Repeated-measures ANOVA was conducted to examine differences in time points within each group. For the intervention group, a significant difference was found between time points [F(2, 48) = 14.8, *p* < 0.001]. Post hoc analysis showed that bedtime was later at baseline than at post-intervention [t(32) = 4.41, *p* < 0.001] and 3 month follow-up [t(32) = 4.02, *p* < 0.001]. Post-intervention and 3 month follow-up were not significantly different from each other. Within the control group, a significant difference was found between time points [F(2, 40) = 7.71, *p* < 0.01]. Bedtime was earlier at baseline than at post-intervention [t(34) = −2.69, *p* < 0.05] and 3 month follow-up [t(34) = −2.94, *p* < 0.01], with no significant difference between post-intervention and 3 month follow-up.

Sleep duration: A significant main effect was found for group [F(1, 66) = 39.17, *p* < 0.001], with longer sleep duration in the intervention group than the control group. A significant main effect was found for time [F(2, 124) = 5.49, *p* < 0.01], such that sleep duration in the 3 month follow-up was shorter than baseline [t(134) = 2.78, *p* < 0.01] and post-intervention [t(134) = 2.86, *p* < 0.01]. Additionally, a significant time X group interaction was found [F(2, 124) = 54.69, *p* < 0.001]. Post hoc analysis showed that groups differed at post-intervention [t(60) = 7.97, *p* < 0.001] and at follow-up [t(51) = 8.8, *p* < 0.001], with longer sleep duration in the intervention group than the control group. Repeated-measures ANOVA showed a significant difference between time points in the intervention group [F(2, 56) = 22.52, *p* < 0.001], with longer sleep duration at post-intervention [t(32) = −5.34, *p* < 0.001] and at 3 month follow-up [t(32) = −5.22, *p* < 0.001] compared to baseline, with no difference between post-intervention and 3 month follow-up. A significant difference between time points was also found in the control group [F(2, 68) = 35.02, *p* < 0.001], with longer sleep duration at baseline compared to post-intervention [t(34) = 5.0, *p* < 0.001] and 3 month follow-up [t(34) = 7.38, *p* < 0.001], and longer sleep duration at post-intervention than at 3 month follow-up [t(34) = 3.84, *p* < 0.001] (Figure 2).

Sleep efficiency: A significant main effect was found for group [F(1, 63) = 7.09, *p* < 0.01], with higher sleep efficiency in the intervention group than the control group. A significant main effect was found for time [F(1.68, 106.18) = 7.29, *p* < 0.01], such that sleep efficiency in the 3 month follow-up was higher than at baseline [t(63) = −3.10, *p* < 0.01] and post-intervention [t(63) = 4.43, *p* < 0.001]. However, sleep efficiency at post-intervention was not different from follow-up [t(63) = 0.57, *p* > 0.05]. Additionally, a significant time X group interaction was found [F(1.68, 106.18) = 27.23, *p* < 0.001]. Post hoc analysis showed that groups differed at post-intervention [t(59.60) = 2.19, *p* < 0.05] and at follow-up [t(63) = 5.90, *p* < 0.001], with higher sleep efficiency in the intervention group than the control group. Repeated-measures ANOVA showed a significant difference between time points in the intervention group [F(1.38, 41.69) = 11.02, *p* < 0.001], with higher sleep efficiency at post-intervention [t(29) = −3.41, *p* < 0.01] and at 3 month follow-up [t(29) = −3.60, *p* < 0.01] compared to baseline, with no difference between post-intervention and 3 month follow-up. A significant difference between time points was also found in the control group [F(1.41, 46.81) = 25.83, *p* < 0.001], with lower sleep efficiency at 3 month follow-up compared to baseline [t(32) = −5.05, *p* < 0.001] and compared to post-intervention [t(32) = −6.06, *p* < 0.001], with no difference between baseline and post-intervention (Figure 2).

### 3.5. SSHS

The following effects were found for the self-reported sleep measures (Table 2):

Weekday bedtime: A significant time X group interaction was found [F(2, 83) =4.13, *p* < 0.05], with no significant post hoc comparisons.

Weekday sleep latency (minutes): A main effect was found for time [F(2, 55) = 9.21, *p* < 0.01]. Sleep latency was shorter at baseline compared to post-intervention [t(86) = −3.3, *p* < 0.01] and 3 month follow-up [t(86) = −3.03, *p* < 0.01], with no difference between post-intervention and 3 month follow-up. A significant time X group interaction was found [F(2, 55) = 5.55, *p* < 0.05], with no significant differences between the groups at any of the time points in the post hoc analysis. Repeated-measures ANOVA showed a significant difference between the time points in the intervention group [F(2, 24) = 10.29, *p* < 0.01]. Post hoc analysis showed that sleep latency was longer at post-intervention [t(23) = −3.43, *p* < 0.01] and at 3 month follow-up [t(23) = −3.04, *p* < 0.01] compared to baseline, but was not different between post-intervention and 3 month follow-up. Within the control group, no differences were found between the time points.

Wake-up time on the weekend: A significant time X group interaction was found [F(2, 80) = 4.09, *p* < 0.05], with no group differences between any of the time points in the post hoc analysis. While there were no significant differences between time points within the intervention group, there was a difference between time points within the control group [F(2, 33) = 4.22, *p* < 0.05]. Wake-up time at post-intervention was later than at 3 month follow-up [t(19) = 2.67, *p* < 0.05].

### 3.6. Electronic Media (EM)

EM outcomes were analyzed separately for the entire day and for the evening before bedtime.

**Figure 3:**
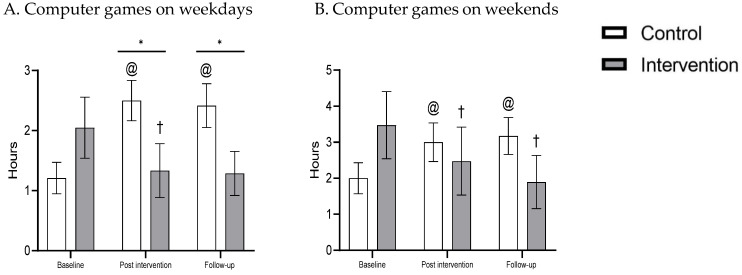
Computer games (hours) weekdays and weekend at baseline, post-intervention and follow-up, intervention and control groups. * *p* < 0.05, † different from intervention baseline, @ different from control baseline.

The following effects were found for EM measures (Table 2 and Figure 3):

Computer games on weekdays—entire day: As a sex difference was found at baseline, sex was included in the model as a covariate. No main effects were found for group and time; however, a main effect was found for sex [F(1, 41) = 6.62, *p* = 0.013], with boys spending more time on the computer than girls, and a time X group interaction was found [F(2, 82) = 14.07, *p* < 0.001], with longer playing time in the control group than the intervention group at post-intervention [t(43) = −2.11, *p* < 0.05] and at three months follow-up [t(43) = −2.17, *p* < 0.05]. Within the intervention group, significant differences were found between time points [F(2, 33) = 3.92, *p* < 0.05], with longer playing time at baseline compared to post-intervention [t(20) = 2.36, *p* < 0.05] and 3 month follow-up [t(20) = 2.05, *p* = 0.053], and no difference between post-intervention and 3 month follow-up. Within the control group, there was a significant difference between time points [F(2, 46) = 12.84, *p* < 0.001], with shorter playing time at baseline compared to the post-intervention [t(23) = −4.74, *p* < 0.001] and 3 month follow-up [t(23) = −5.02, *p* < 0.001], and no difference between post-intervention and 3 month follow-up. No time X sex or time X group X sex interactions were found.

Evening: No group differences were found at any of the time points. Evening data showed a main effect for time within the intervention group [χ^2^(2) = 8.33, *p* < 0.05, partial η2 = 0.21]. Compared to baseline, participants spent less time playing computer games at post-intervention [Z = −2.12, *p* < 0.05] and at 3 month follow-up [Z = −2.07, *p* < 0.05]. No difference was found between post-intervention and 3 month follow-up. No main effect was found for the control group.

Computer games on weekend—entire day: While no main effects were found for group and time, a main effect for sex was found [F(1, 38) = 8.65, *p* = 0.005], with boys spending more time on the computer than girls.. A significant time X group interaction was found [F(2, 76) = 12.55, *p* < 0.001]. Post hoc analysis showed no group difference at any of the time points. Within the intervention group, there was a significant difference between time points [F(2, 36) = 4.68, *p* < 0.05], with longer playing time at baseline compared to post-intervention [t(18) = 2.47, *p* < 0.05] and 3 month follow-up [t(18) = 2.63, *p* < 0.05], and no difference between post-intervention and 3 month follow-up. Within the control group, there was also a significant difference between time points [F(2, 44) = 10.45, *p* < 0.001], with less playing time at baseline compared to post-intervention [t(22) = −3.74, *p* < 0.01] and 3 month follow-up [t(22) = −5.24, *p* < 0.001] and with no difference between post-intervention and 3 month follow-up. No time X sex or time X group X sex interactions were found.

Evening: No group differences were found at any of the time points. Separate analyses of the evening data revealed a significant effect within the intervention group [χ^2^(2) = 8.24, *p* < 0.05, partial η2 = 0.27]. Compared to baseline, the participants spent less time playing computer games at 3 month follow-up [Z = −2.45, *p* < 0.05]. No difference was found between baseline and post-intervention, and between post-intervention and 3 month follow-up. No main effect was found for the control group.

TV time/internet use: No main effects or interactions were found for TV time or internet use, either for the entire day or for the evening, during weekdays or weekends.

## 4. Discussion

This study evaluated the effectiveness of a parent-focused intervention aimed to promote healthy sleep patterns and controlled exposure to EM in young adolescents. Group differences found in this study may be attributed to the treatment modality, rather than intervention materials and content. Golan’s Model [21] views the parents as the sole agents of change in the lives of their children, and focuses on the power of personal example and environmental changes. This model has been found to be effective in the field of eating disorders, but is implemented here for the first time in the field of sleep. On the theoretical level, this research supports and further contributes to Golan’s Model. Regarding practical application, the findings show that programs tailored for parents can influence sleep patterns in young adolescents.

Adolescents in the study sample slept an average of 8 h or more on weekdays. These results are consistent with other studies and do not meet the recommended sleep time for this age group [7,13,23]. Adolescents reported that they spent an average of almost four hours using media daily on weekdays and almost four and a half hours on weekends. These findings agree with previous surveys in which Israeli adolescents were defined as “heavy users” of media [24,25]. After intervention and 3 month follow-up, objective (actigraphy) measures showed that the intervention group, on average, went to sleep approximately 22 min earlier, slept on average 30 min more than baseline and showed higher sleep efficiency in the 3 month follow-up than at baseline (a 1.897% increase), and at post-intervention (a 2.21% increase). These findings are consistent with Short et al. (2019) [9], who found that adolescents whose parents set their bedtimes went to bed approximately 45 min earlier and obtained 45 min more sleep, compared to adolescents whose parents are not involved [9].

Over a 3 month period, between baseline and follow-up, the control group went to sleep approximately 22 min later, slept on average 37 min less and showed a decrease in sleep efficiency by 5%. Age-related delay in adolescent bedtime and consequent reduction is sleep duration is well documented [26,27,28,29] and may at least partially explain these changes in the control group. Another possible explanation may be the different timing of measurements within the school year between the intervention and control groups. Although measurements for both groups were collected during the autumn–winter standard time, it is possible that workload was increased during the later months of the school year. High school workload in adolescents has been implicated in delayed bedtime and curtailed sleep [30].

At the post-intervention and 3 month follow-ups, the intervention group was found to have reduced time playing computer games, during the entire day and in the evening, on weekdays on average by half an hour, and by an hour on weekends. The control group was exposed to more EM: playing computer games increased by one hour on weekends and by more than one hour on weekdays. These results are similar to a Swedish study [31] that demonstrated a positive correlation between age and EM use. Thus, children aged 6–10 years old estimated the time they spent watching TV as 1 h a day, whereas adolescents aged 14–16 estimated that they spent 1½ h a day watching TV [31]. Parental involvement moderated the negative relationship between age, sleep, and EM.

Most parents reported positive feedback and high levels of satisfaction from the meetings. They noted the friendly, non-judgmental atmosphere, and the ability not only to acquire knowledge, but also to consult for the first time with experts in the area and to interact with other parents. The group discussions made them realize that they were not alone in the dilemmas that they faced with their teens. In a meta-analysis examining whether parent training interventions are effective in reducing ADHD symptoms and associated problems in ADHD-diagnosed children between the ages of 5 and 18, the authors noted the positive effects of parental training not only on the behavior of children with ADHD, but on parents’ confidence in implementing the interventions with their child [32].

Parents tend to show a high level of cooperation, in contrast to recruitment difficulties and low motivation with adolescents [10,13]. In addition, parents’ involvement was found to improve sleep patterns and lower depression and suicidal ideation rates of their children [22]. Therefore, future interventions should focus on providing parents with tools to help motivate their children, for the benefit of the entire family.

Limitations of this study: This study has some notable limitations. Follow-up was only at three months post-intervention, and thus we could not assess whether the improvements were maintained beyond that time. Nevertheless, other studies have also used a similar time frame: 6 weeks [10], 6 weeks [15] and 6, 12, and 20 weeks [15]. The intervention did not address the motivational component from the adolescents’ point of view. Nevertheless, findings clearly demonstrated positive changes in sleep patterns and media habits, suggesting that parental involvement is a crucial ingredient in modifying behavior. The intervention did not address the use of mobile phones; however, this study took place 6–8 years ago, when few adolescents in this age group had mobile phones. Although the intervention did lead to changes in sleep and media use, changes in the home environment were not quantified, and therefore this study cannot specify which changes occurred in the participants’ homes. Additionally, the division into groups was not random; rather, there were two recruitment periods, first for the intervention group and then for the control group. Finally, the study sample included adolescents within a narrow age range (10–12 years), from rural communities in northern Israeli households with two parents and with an above-average socioeconomic status. Therefore, findings may not be easily generalized to older adolescents, urban settings, single-parent households and/or lower SES. We found a considerable gap between Actigraphy and self-report measures. Follow-up studies should check the size of the gap.

Follow-up studies will be able to track and execute the proposed model while randomly dividing it. Additionally, future interventions may focus on addressing parents and providing them with tools aimed to motivate their children and reduce the technological gap between children and parents. Today, mobile phones are widely used by most adolescents in this age group. Future interventions must address the ongoing changes in new media technologies and platforms.

## 5. Method

### 5.1. Study Design

This prospective study was a comparative group design that included an intervention group and a control group.

### 5.2. Study Population

The total sample size was determined to be 70, based on a priori power analysis using G*Power software (v3.1). We used a moderate effect size based on previous finding in the literature [15], at an alpha error probability of 0.05 and a power of 0.95 to detect an existing effect. Participants included 70 dyads of young adolescents in elementary school (5th and 6th grades) and their parents (68 mothers and 2 fathers) in northern Israel. According to the inclusion criteria, the adolescents were boys and girls aged 10–12, healthy (i.e., with no particular physical or mental health problems), fluent Hebrew speakers, from families with both parents living in the same household and studying in normative classroom settings. Parents were mothers or fathers, generally healthy, and fluent Hebrew speakers, who reported concerns regarding their adolescent child’s sleep habits and/or EM exposure habits. Families living in households without EM devices were excluded.

### 5.3. Recruitment

This study took place from 2012 to 2014. Advertisements at schools and in the education departments of municipal councils invited parents to participate in a clinical trial aimed at improving the sleep habits of their young adolescents. Interested parents were recruited after information about this study was provided. The division into groups was not random; rather, there were two recruitment periods, first for the intervention group and then for the control group. Recruitments were performed consecutively, first for the intervention group and subsequently for the control group. A home visit was scheduled for parents and adolescents who were eligible (see section on study population) and who agreed to participate in this study. At that time, both parents and adolescents signed informed consent forms and were provided with the study materials for baseline assessments.

### 5.4. Measurements Collected from Adolescents

The School Sleep Habits Survey (SSHS) [33]; Hebrew translation [2] includes demographic data, sleep patterns including bedtime (hrs:min), sleep latency (min), wake-up time (hrs:min), and total sleep duration (hrs), both on weekdays and weekends.

Modified Electronic Media Questionnaire (EMFQ) [34]: To assess EM exposure (including television, computer games, and internet use), the EMFQ was translated based on accepted guidelines and modified for the sake of simplicity. Adolescents reported whether they had a television/computer in their bedroom. They reported exposure to television, computer games and internet (in hours) during different periods of the day, over a 7 day period. For the purposes of this study, the day was measured from 6 am until bedtime, separated into early morning (6 a.m.–8 a.m.), morning (8 a.m.–2 p.m.), afternoon (2 p.m.–5 p.m.), evening (5 p.m.–8 p.m.), and night (8 p.m.–bedtime). Viewing durations were averaged to form overall weekday and weekend measures of television and computer exposure.

Actigraphy: Sleep patterns were assessed objectively by a wrist-worn actigraph (AMI, NY) on subjects’ non-dominant wrists for 5 nights on school nights only. Data were analyzed using the Sadeh algorithm [35]. Actigraphy measures were: bedtime (hh:mm), wake time (hh:mm), sleep duration (hours), sleep efficiency (percentages), and sleep latency (minutes).

Sleep diary: Sleep diaries were used as supplemental information for analyzing the actigraphy data. The diary included the following measures: Did you sleep during the day? Specify the level of fatigue today? 1 = Very alert to 5 = Very tired. Did you take any you turn off the light? how long it took you to fall asleep? Number and duration of awakenings at night, waking time in the morning? What time did you get out of bed? How long did you sleep? What was your sleep quality? 1 = best–5 = worst.

### 5.5. Procedure

The three waves of data collection were baseline, post-intervention, and 3 month follow-up (3 months between baseline and post-intervention and 3 months between post-intervention and follow-up), which were carried out by the research team. All of the data were collected during the same school year at standard times between November and March. In each wave, adolescents completed the modified SSHS and EMFQ, and wore an actigraph with a supplemental sleep diary.

**Figure 4:**
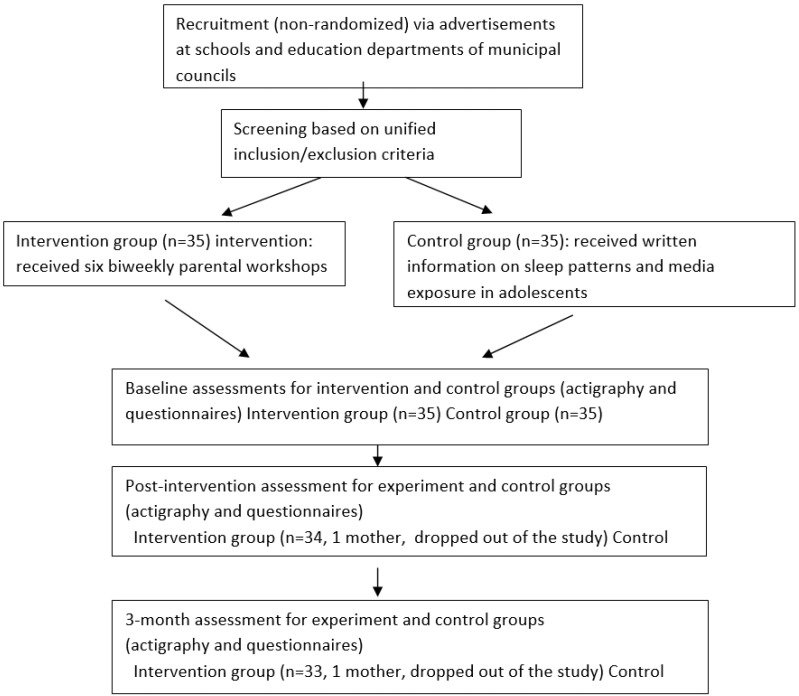
Flowchart of study procedures.

The intervention began immediately following baseline data collection. Parents in the intervention group participated in a six-session workshop intervention, with sessions occurring every two weeks over a 3 month period (see Flowchart in Figure 4). Parents in the control group received written information by mail on the same themes that were presented in the workshop. Repeated assessments were performed one-week post-intervention and at a 3 month follow-up. Parents were scheduled for home visits to provide and collect study materials for their adolescents.

### 5.6. Workshop

The six-session workshop provided educational materials relating to three main subjects: (1) the physiological, psychological, and social changes that take place during adolescence; (2) the importance of sleep, i.e., adequate sleep, and environmental factors such as media use that affect sleep; and (3) the authoritative parenting style, characterized by openness, understanding, and setting rules, as an effective style for implementing sustainable changes. The workshops were conducted at Emek Yezreel Academic College during evening hours. The workshop was facilitated by an expert group facilitator (co-author OF-B), and experts in sleep in adolescents (co-authors OT, TS) served as moderators. The same experts took part as facilitators in all intervention groups. The workshops sessions included 10–11 parents per group (one parent from each family, mostly the mother); the children did not participate in the workshops. The intervention sessions included information on the relevant topics, as well as a conversation and consulting between the participants based on their experience.

Parents were encouraged to share their experiences related to their child’s bedtime and screen exposure habits, to work at providing solutions for difficult situations such as adolescent resistance, and to discuss ways to create motivation for change among the adolescents (see Table 3). Written information was sent by mail to the parents in the control condition.

### 5.7. Statistical Analysis

Descriptive statistics, including frequencies, means, and standard deviations, were calculated for baseline, post-intervention, and 3 month follow-up measurements. Analyses of the data were conducted within individual subjects (pre- and post-intervention and at follow-up) and between groups (intervention vs. control). Repeated-measures ANOVA with t-test post hoc analyses were performed to assess changes in adolescent sleep and EM patterns over the entire day, controlling for sex where sex differences were found (computer games only). Friedman tests with Mann–Whitney U and Wilcoxon signed-rank post hoc analyses were performed to assess changes in EM patterns in the evening (8 p.m.–bedtime) due to non-normal distribution. Effect sizes were calculated, for time, group and time*group, using partial eta-squared.

## 6. Conclusions and Implications in the COVID-19 Era

This research is the first to demonstrate the effectiveness of an intervention program for the improvement of sleep patterns and controlled use of EM with parents as the sole agents of change. The study findings support Golan’s Model [21] and demonstrate that the model may be used to address other areas of health in adolescents in addition to eating habits. The intervention program partially improved sleep patterns (bedtime, sleep duration and sleep efficiency) and EM (computer games). All parents were cooperative, demonstrating high feasibility. Parents in the control group did not create positive change, suggesting that written material is not sufficient, whereas group support may enable parents to be more active in order to create change.

The current COVID-19 pandemic created changes in health-related lifestyle habits due to lockdowns and social distancing. An online survey of adults aged 18–75 years old demonstrated that both positive and negative changes in lifestyle habits, e.g., cigarette smoking, sleep and physical activity, were associated with higher psychological distress (depression, anxiety, and stress), and that COVID-related stressors (e.g., financial problems and increased work and family responsibilities, uncertainty about the future) moderated these associations [36]. These findings may be amplified in youth and suggest that the maintenance of routine health behaviors is crucial during major stressful life events.

Furthermore, social media plays a huge role in the lives of children and teenagers, especially during the COVID-19 pandemic. Recent research confirms that the use of social media by high school students contributes significantly to their well-being during the pandemic inasmuch as it fosters interpersonal communication, supports personal interests, and is used as a tool for study and entertainment [37]. However, authors acknowledge that the relationship between social media use and well-being is controversial, with several lines of evidence suggesting negative mental health and well-being outcomes due to social media use.

These and other studies stress the paramount importance of maintaining healthy lifestyle habits, including well-balanced sleep and media exposure, in youth during the COVID-19 era. Accordingly, our present findings encourage parents to be proactive and involved in enabling their children to achieve and maintain proper health habits, including beneficial sleep patterns, and supervised media use.

## Figures and Tables

**Table 1:** Means (SD/df) and partial η2 of sleep measures (actigraphy and adolescent reports-SSHS) at baseline, post-intervention and 3 month follow-up, by time, group, and time*group interaction.

Intervention				Control					
	Baseline	Post-Intervention	Follow-Up	Baseline	Post-Intervention	Follow-Up	Time F, (df) Partial η2	Group F, (df) Partial η2	Time × Group F, (df) Partial η2
Actigraphy									
Bedtime (hours)	22:00 (0:28)	21:38 (0:25)	21:36 (0:28)	22:21 (0:50)	22:43 (0:35)	22:49 (0:42)	0.24 (2,84) 0.003	54.54 *** (1, 66) 0.452	18.62 *** (2,84) 0.220
Wake-up time (hours)	6:36 (0:21)	6:42 (0:21)	6:42 (0:20)	6:34 (0:30)	6:38 (0:28)	6:31 (0:29)	2.21 (2,132) 0.32	0.95 (1,66) 0.014	1.75 (2,132) 0.26
Sleep duration (hours)	8:31 (0:38)	9:01 (0:30)	9:04 (0:31)	8:23 (0:59)	7:46 (0:45)	7:21 (1:01)	5.49 ** (2,124) 0.077	39.17 *** (1,66) 0.372	54.69 *** (2,124) 0.453
Sleep efficiency (percentage)	93.11% (3.95%)	95.08% (2.76%)	95.32% (2.45%)	93.29% (4.07%)	93.26%(3.88%)	90.61%(3.76%)	7.29 ** (1.68, 106.18) 0.10	7.09 ** (1, 63) 0.10	27.23 *** (1.68, 106.18) 0.30
Sleep latency (minutes)	00:33 (00:23)	00:31 (00:18)	00:28 (00:15)	00:34 (00:23)	00:29 (00:18)	00:29 (00:15)	2.76 (2,105) 0.042	0.00 (1,63) 0.000	0.39 (2,105) 0.006
SSHS									
Bedtime (hours)—weekdays	21:41 (0:35)	21:47 (0:30)	22:04 (0:56)	21:51 (0:40)	21:54 (0:46)	21:39 (0:58)	0.36 (2,83) 0.008	0.08 (1,45) 0.002	4.14 * (2,83) 0.084
Bedtime (hours)—weekends	23:03 (0:54)	23:17 (0:44)	23:23 (0:58)	23:33 (1:36)	23:41 (1:40)	23:27 (1:58)	0.48 (2,78) 0.022	0.37 (1,42) 0.017	2.11 (2,78) 0.035
Wake-up time (hours)—weekdays	6:45 (0:35)	6:53 (0:21)	6:37 (1:22)	6:45 (0:31)	6:47 (0:28)	6:47 (0:33)	0.60 (2,57) 0.013	0.14 (1,45) 0.000	0.68 (2,57) 0.015
Wake-up time (hours)—weekends	9:19 (1:17)	9:34 (1:23)	9:40 (1:39)	9:57 (2:01)	10:13 (1:37)	9:27 (2:19)	2.01 (2,80) 0.045	0.56 (1,43) 0.013	4.09 * (2,80) 0.087
Sleep latency (minutes)—weekdays	00:15 (00:15)	00:35 (00:29)	00:34 (00:31)	00:20 (0:18)	0:23 (0:19)	00:21 (00:18)	9.22 ** (2,55) 0.180	1.13 (1,42) 0.026	5.55 * (2,55) 0.117
Sleep latency (minutes)—weekends	00:15 (00:17)	00:19 (00:16)	00:17 (00:17)	00:13 (00:13)	00:15 (00:13)	00:16 (00:17)	0.48 (2,88) 0.011	0.37 (1,44) 0.008	0.21 (2,88) 0.050
Sleep duration (hours)—weekdays	8:19 (1:36)	8:33 (0:52)	8:31 (1:13)	8:44 (1:06)	8:24 (2:17)	8:57 (1:12)	0.87 (2,90) 0.019	0.50 (1,45) 0.011	1.15 (2,90) 0.025
Sleep duration (hours)—weekends	9:35 (1:80)	9:30 (1:31)	9:48 (1:34)	10:06 (1:30)	9:36 (2:42)	10:10 (1:28)	1.09 (2,68) 0.026	0.56 (1,41) 0.014	0.28 (2,68) 0.007

* *p* ≤ 0.05; ** *p* ≤ 0.01; *** *p* ≤ 0.001.

**Table 2:** Means (SD/df) and partial η2 of EM measures at baseline, post-intervention and 3 month follow-up, in hours, by time, group, and time*group interaction.

	Intervention			Control					
	Baseline	Post tx	Follow-Up	Baseline	Post-Intervention	Follow- Up	Time F, (df) Partial η2	Group F, (df) Partial η2	Time × Group F, (df) Partial η2
TV time—Weekdays (hours)	4.32 (2.63)	04.48 (2.53)	04.44 (2.63)	3.30 (1.55)	4.13 (1.98)	4.42 (1.62)	1.28 (2,92) 0.027	0.89 (1,46) 0.019	2.28 (2,92) 0.040
TV time—Weekends (hours)	5.048 (3.60)	5.35 (4.41)	5.056 (3.65)	3.56 (1.82)	4.17 (1.85)	4.87 (1.84)	1.25 (2,81) 0.028	2.93 (1,44) 0.062	0.94 (2,81) 0.021
Internet use—Weekdays (hours)	2.23 (1.97)	1.68 (2.30)	2.54 (1.82)	2.45 (2.48)	2.64 (1.62)	3.13 (2.53)	1.77 (2,57) 0.040	1.52 (1,42) 0.035	0.47 (2,57) 0.011
Internet use—Weekends (hours)	3.57 (2.74)	2.93 (2.62)	3.00 (2.57)	2.55 (2.22)	3.45 (2.26)	3.64 (2.56)	0.27 (2,50) 0.008	0.00 (1,34) 0.000	3.49 (2,50) 0.093
Computer Games—Weekdays (hours) #	2.05 (2.33)	1.33 (2.06)	1.29 (1.68)	1.21 (1.28)	2.50 (1.64)	2.42 (1.79)	1.22 (2,82) 0. 028	2.31 (1,41) 0.053	14.15 *** (2,82) 0.255
Computer Games—Weekends (hours) ##	3.47 (4.07)	2.47 (4.11)	1.89 (3.21)	2.00 (2.07)	3.00 (2.56)	3.17 (2.46)	0.1 (2,76) 0. 004	0.66 (1,38) 0.017	12.55 *** (2,76) 0.248

*** *p* ≤ 0.001. # Main effect for gender was found [F(1, 41) = 6.62, *p* = 0.013, partial η2 = 0.139]. ## Main effect for gender was found [F(1, 38) = 8.65, *p* = 0.005, partial η2 = 0.185].

**Table 3:** Thematic intervention sessions.

Session	Themes	Home Practice Activities
1	Preliminary meeting, introductions between group members, explanation of this study’s design and aims.	
2	Physiological, psychological, and social changes in adolescence. The importance of sleep: adequate sleep, REM sleep, and environmental factors that affect sleep, including media.	During the day:
		- Reduce media exposure - Remove media devices during meals - Offer children more outdoor activities and social interaction
		Sleep hygiene:
		- Sleep only in a quiet and dark the bedroom. - Avoid caffeine in the evening. - Avoid sports/athletic activities 2 h before sleep. - Avoid media devices 1 h before sleep. - Take media devices out of the bedroom. - Turn off or silence mobile phones and remove from bedroom before going to sleep. - Open shutters in the morning. - Bedtime no later than 22:00–23:00. - On weekdays, go to sleep at the same time every night. - Maintain regular sleep times on weekends and holidays. - Avoid sleep during the day.
3	Part 1: Review of the 12 rules. Parents discuss how and whether they managed to implement the rules. Part 2: Authoritative parenting style: understanding the importance of setting rules.	
4	How to be an authoritative parent: dealing with resistance and creating motivation for change among teens.	Adopting the authoritative parenting style:
		- Set clear rules while avoiding conflict. - Explain the rationale beyond every rule. - Mention house rules several times a day until the behavior is internalized. - Give sanctions when necessary.
5	Review of the content of the last session. Parents’ share how and whether they managed to set new rules regarding media exposure and sleep patterns at home, and the instructor gives tips for better results.	
6	Summarize the content of all sessions and repeat all the practice activities.	
